# Flexible and Lightweight Devices for Wireless Multi-Color Optogenetic Experiments Controllable via Commercial Cell Phones

**DOI:** 10.3389/fnins.2019.00819

**Published:** 2019-09-06

**Authors:** Philipp Mayer, Nandhini Sivakumar, Michael Pritz, Matjia Varga, Andreas Mehmann, Seunghyun Lee, Alfredo Salvatore, Michele Magno, Matt Pharr, Helge C. Johannssen, Gerhard Troester, Hanns Ulrich Zeilhofer, Giovanni Antonio Salvatore

**Affiliations:** ^1^Electronics Laboratory, ETH Zurich, Zurich, Switzerland; ^2^Institute for Integrated Circuits, ETH Zurich, Zurich, Switzerland; ^3^Institute of Pharmacology and Toxicology, University of Zurich, Zurich, Switzerland; ^4^Department of Mechanical Engineering, Texas A&M University, College Station, TX, United States; ^5^Sensor ID, Campochiaro, Italy; ^6^Salvatore Optopharma, Zurich, Switzerland

**Keywords:** wireless, flexible electronics, optogenetics, *in vivo* experiments, nociception, pain, channelrhodopsin, archaerhodopsin

## Abstract

Optogenetics provide a potential alternative approach to the treatment of chronic pain, in which complex pathology often hampers efficacy of standard pharmacological approaches. Technological advancements in the development of thin, wireless, and mechanically flexible optoelectronic implants offer new routes to control the activity of subsets of neurons and nerve fibers *in vivo*. This study reports a novel and advanced design of battery-free, flexible, and lightweight devices equipped with one or two miniaturized LEDs, which can be individually controlled in real time. Two proof-of-concept experiments in mice demonstrate the feasibility of these devices. First, we show that blue-light devices implanted on top of the lumbar spinal cord can excite channelrhodopsin expressing nociceptors to induce place aversion. Second, we show that nocifensive withdrawal responses can be suppressed by green-light optogenetic (Archaerhodopsin-mediated) inhibition of action potential propagation along the sciatic nerve. One salient feature of these devices is that they can be operated via modern tablets and smartphones without bulky and complex lab instrumentation. In addition to the optical stimulation, the design enables the simultaneously wireless recording of the temperature in proximity of the stimulation area. As such, these devices are primed for translation to human patients with implications in the treatment of neurological and psychiatric conditions far beyond chronic pain syndromes.

## Introduction

Chronic pain is a highly debilitating condition that affects about 20% of the general population (Breivik et al., 2006). It involves both enhanced input from peripheral nociceptors and altered central pain processing. In many patients, chronic pain is resistant to current medications, likely because highly selective targeting of specific signaling pathways does not adequately take into account the complexity of chronic pain syndromes. Optogenetics provides an alternative approach. It employs the transgenic expression of light-sensitive ion channels or pumps to tightly control the activity of certain neurons or neuronal projections through light (Williams and Deisseroth, 2013). Dependent on the specific type of opsin employed, light stimulation can be used to either activate or inhibit neuronal activity (Nagel et al., 2003; Chow et al., 2010). Optogenetic devices implanted on top of a peripheral nerve or on the dorsal surface of the spinal cord allow the manipulation of excitatory input from peripheral nociceptors, or of central neurons or fiber tracts located superficially in the spinal cord. While such manipulations can in principle be achieved with fiber optic based solutions (Bonin et al., 2016), therapeutic application and use in sophisticated rodent behavioral paradigms of analgesia would benefit from fully implantable and durable wireless electronic systems ideally carrying more than one light source for inhibition/excitation of neurons that can be individually controlled on an on-demand basis (Kwon et al., 2015; Montgomery et al., 2015; Rossi et al., 2015).

Conventional printed electronics boards are rigid and bulky and often protrude several millimeters under the skin. Their mechanical format hardly adapts to the soft mechanics of the tissues and can induce damage during prolonged use. Moreover, the aforementioned mismatch precludes device immobilization and, consequently, the efficient delivery of the optical stimuli in the region of interest. Recent advances in material science and micro-technology have enabled the integration of high performance and miniaturized electronic chips on soft polymeric substrates so as to confer physical properties, such as thickness and Young’s modulus, that resemble those of biotissues (Kim et al., 2013; Park et al., 2015; Gutruf and Rogers, 2018). Such devices can laminate onto the spinal cord, operate in wireless mode, and provide the desired optical power (1–20 mW/mm^2^) for optogenetics (Shin et al., 2017; Gutruf and Rogers, 2018).

This study reports a novel and advanced design of battery-free, flexible, and lightweight devices, equipped with one or two miniaturized LEDs powered by resonant magnetic coupling that can be individually controlled in real time. Here, we demonstrate that these devices can be implanted on top of the spinal cord or near a peripheral nerve to control the activity of nociceptors and to evoke or suppress nociception. Importantly, these devices can be controlled via modern tablets and smartphones without bulky and complex lab instrumentations. As such, these devices are primed for translation to human patients with implications in the treatment of neurological and psychiatric conditions far beyond chronic pain syndromes.

## Results

### 1-LED Device

**FIGURE 1:**
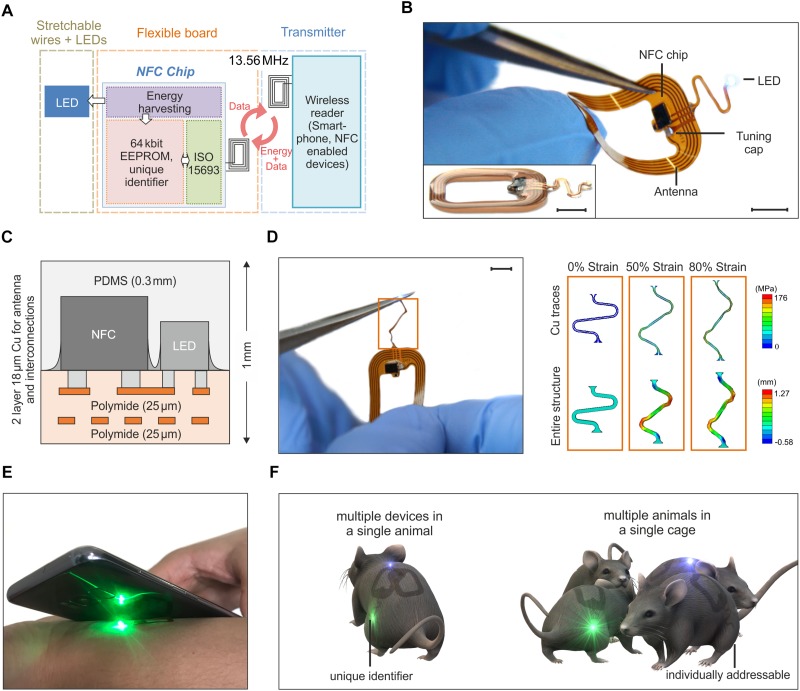
Overview of the assembly and functionalities of the device. **(A)** Schematic of the building blocks of the device. Resonant inductive coupling and Near Field technology chip operating at 13.56 MHz wirelessly power the LED. **(B)** Device operating during bending tests. The antenna is 1.9 cm × 1.4 cm large and it is formed by 5 Cu turns with a thickness of 18 μm and a pitch of 250 μm. The interconnection between the electronics and the LED has an open and S-shaped design which enables out-of-plane displacement during implantation (scale bar 5 mm). The inset shows a side-view of the device (scale bar 5 mm). **(C)** The flexible electronic board is formed by two metal layers encapsulated by thin polyimide and PDMS films for a thickness of 1 mm at the location of the chips and 0.4 mm at the interconnections. **(D)** Stress-strain simulation of the interconnections between the LED and the antenna (scale bar 5 mm). The top row shows the maximum principal in-plane stress in the Cu. The bottom row shows the out of plane displacement of the entire structure. The simulation results show that the Cu layer of the interconnect did not yield until a level of –80% global strain (for a yield strength of Cu of 110 MPa). **(E)** Device activated and operated by a mobile phone. **(F)** The NFC chip has a unique identification code, that allows for activating and controlling multiple devices. Such functionality offers the possibility of running experiments with multiple optogenetic implants in a single mouse or/and experiments with multiple mice.

Figure 1 illustrates the key features of a thin, flexible wireless optoelectronic system that exploits inductive resonant coupling and Near Field Communication (NFC) technology to power and control a surface mounted miniaturized LED. Figure 1A presents the block diagram of the functional components. A wireless link at 13.56 MHz is established by magnetic induction between coils associated with the device and an external reader (i.e., any NFC-enabled smartphone, tablet, etc.) and enables power delivery to an ISO 15693 based NFC chip, which monolithically integrates 64k-bit data storage and energy harvesting capabilities. The latter provides DC power to the LED (OSRAM LT QH9G for green, LB QH9G for blue, 1 mm × 0.4 mm, thickness ∼0.4 mm). Among Radio Frequency (RF) power transfer approaches, magnetically coupled resonators have shown the potential to deliver power with more efficiency than far-field approaches, and at longer ranges than traditional inductively coupled schemes (Kurs et al., 2007; Cannon et al., 2009; Low et al., 2009). Moreover, power transmission in the near field regime demonstrates little sensitivity to the presence of objects or physical obstructions, including those environments made of metals or with significant water content. Reliable operation is, in fact, even possible underwater and/or through metallic cages and/or plates with minimal requirements in RF optimization and tuning. These are attractive characteristics to power implants in freely moving animals for *in vivo* optogenetics experiments.

The device incorporates various functional layers (copper metallization of ∼18 μm), barrier films [polyimide and poly(dimethylsiloxane)], and active components (surface-mounted chips and LEDs) fabricated on a substrate of polyimide (25 μm thickness) in a planar geometry to facilitate processing by conventional manufacturing techniques (Figure 1B). All metal traces include encapsulating layers of polyimide above and below to physically and electrically insulate the copper and to place it near the neutral mechanical plane (Figure 1C). The rectangular loop antenna (outer dimensions of 14 mm × 19 mm) exploits 5 turns of copper lines with a pitch of 250 μm and widths and thicknesses of ∼250 μm and 18 μm, respectively. The antenna has an inductance and resistance of 684 nH and 1 Ω at 13.56 MHz, respectively, and an equivalent Q factor of 58.5. Here, a capacitor (174 pF) provides impedance matching with the input impedance of a miniaturized Quad-flat-no-leads-package NFC chip (ST M24LR, 2.38 mm × 2.38 mm, thickness ∼0.5 mm). An open layout design of the interconnections between the electronics and the LED allows out-of-plane motion during manipulation and implantation. Additional information about the circuit design, the chip components and the final appearance of the devices appears in Supplementary Figures S1, S2. After encapsulation with a uniform layer of poly(dimethylsiloxane) (PDMS, ∼300 μm), the maximum thickness of the device is 1 mm (at the location of the chips, i.e., NFC and LED); the minimum thickness is 0.4 mm at the position of the coil and associated interconnect wiring. The overall dimension is 2.5 cm × 1.5 cm. With thin elastomeric substrates and encapsulating layers, these layouts enable large bending deformations of the antenna. Finite element simulations of the mechanical response during stretching of the interconnection between the antenna and the LED provide a quantitative analysis of the stress in the structure. The results, shown in Figure 1D, indicate that stress-concentrating regions exist at the ends (i.e., near the antenna and LED) and at the bends (i.e., near the arcs). To improve this design, those regions should be addressed first. Generally, decreasing the in-plane width of the serpentine relative to the in-plane length and/or decreasing the thickness of the layers will improve stretchability by enabling more out-of-plane deformation at lower corresponding stresses. Additionally, to find when plastic deformation occurred in the composite, a yield strength of Cu of 80 MPa was considered as a representative value (Zhang et al., 2007). Using this value, the finite element simulations show that the Cu layer of the interconnect will not yield until a level of ∼40% global strain. As such, beyond its mere stretchability, we also expect that this design will enable repeated stretching, twisting, etc. without fatigue failure, since plastic deformation of the Cu traces will not occur unless global strains exceed 40% (according to the simulations). These miniaturized dimensions, the lightweight construction (∼100 mg), and the mechanical flexibility represent attractive characteristics as a versatile platform for wireless delivery of light to organs and tissues in freely moving animals.

Devices with similar formats and operating principles have been previously reported in literature. Rogers’ group proposed RF (Shin et al., 2017) and UHF (Park et al., 2015) powering of the devices with designs which include rectification of the AC magnetic field and separate independent antennas to utilize multiple LEDs (Park et al., 2016). Here, however, the introduction of a NFC chip and, in a second improved version presented later in the paper, of a micro-controller enables new functionalities which have not been explored before in flexible wireless devices for applications in optogenetics, such as simultaneously stimulation and recording of the temperature. Primarily, the use of a NFC chip offers the opportunity to interface the implant with a mobile phone and, eventually, to facilitate the translation of this technology to human patients (Figure 1E). Moreover, beyond the functionalities demonstrated in this work, our design opens avenues toward performing new sets of experiments to study the behavior of freely moving animals. Examples of experiments include the use of multiple implants in one single mouse or single implants into multiple mice (Figure 1F) with the ability, in both cases, to individually and selectively control the device owing to the unique electronic identification of the chip.

**FIGURE 2:**
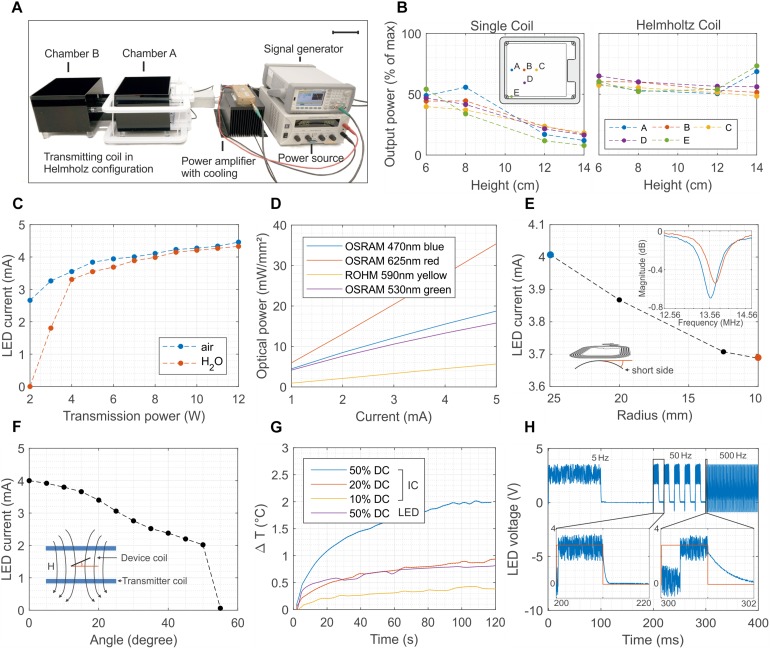
Wireless powering set-up and 1-LED device performance. **(A)** Electronic set-up to wirelessly power the implants. The signal of a double-channel signal generator is amplified and transmitted to the Helmholtz coils. The transmitted power can be as high as 12 W, and it oscillates at 13.56 MHz with a carrier pulse width modulated at a frequency ranging from 1 to 100 Hz (scale bar 10 cm). **(B)** Distribution of the power for one and two coils arranged in Helmholtz configuration. In the latter case, the power has a uniformity of about 10 % across the vast majority of the cage. **(C)** Dependence of the LED current as a function of the transmitted power in both air and water environments The implant is placed in the middle of the cage and perpendicular to the external magnetic field. Water absorption at 13.56 MHz is negligible for transmitted power higher than 4 W. **(D)** Optical power density as a function of the biasing current of four different surface-mounted LEDs. The device combined with the set-up is able to reach optical power density larger than 10 mW mm^–2^ for green and blue LEDs. **(E)** Current of the LED when the flexible antenna of the device is bent from a 25 to a 10 mm radius. Despite of the shift toward right of the resonant frequency, as consequence of the reduced equivalent inductance, the device continues to work even at 10 mm bending radius. **(F)** Dependence of the biasing current of the LED as function of the tilting angle between the antenna of the device and the external magnetic field. The device turns off for angles greater than 50°. **(G)** Temperature increase measured *in-vivo* with two NTCs placed above the NFC chip and the LED. The temperature increase remains below 1°C for 12 W as transmitted power and for 20% duty cycle. **(H)** Switching behavior of the device at 5, 50, and 500 Hz. The maximum switching speed is about 2 kHz and it is limited by the set-uptime of the NFC chip.

Typical *in vivo* optogenetic experiments require stable optical illumination, independent of the posture of the experimental animal and of its position in a test cage. To allow sufficient tissue penetration, the power should be greater than 5 mW mm^–2^, with pulse light modulation at frequencies as high as 1 kHz. Another critical prerequisite is that the temperature increases in the tissue surrounding the device must kept small enough (less than 1 K) to avoid thermal damage. Wireless operation should ensure similar performance in freely moving mice. Figure 2 summarizes the details of the wireless set-up used for the *in vivo* tests and the performance of the 1-LED device. A double channel signal generator and a RF amplifier power two transmission coils which are connected in Helmholtz configuration and which illuminate only one of the two cages used in the experiments (Figure 2A and Supplementary Figure S3 show an optical image and the schematic of the set-up, respectively). The generated magnetic field has a RF carrier wave at 13.56 MHz to interface the NFC chip and a pulse width modulation (PWM) that controls the switching of the LEDs. Figure 2B illustrates the normalized power received by the device at various locations in the cage. For the single transmitter coil, the power drastically decreases with increasing separation distance between the transmitter loop and the device antenna, as a consequence of the exponential decrease of the magnetic field. By comparison, the Helmholtz-coil configuration ensures a much more uniform field in both the in-plane and out-of-plane directions. The experimental data shows a power uniformity in the range of 10% in the xy plane when the device is placed at about 7 cm from the top and bottom so as to be in the middle of the Helmholtz coils (Supplementary Figure S4 shows the in-plane mapping of the power at different heights).

The power received by the device is proportional to the flux of the magnetic field through the surface of its antenna (Feynman et al., 1963) and thus depends on the transmitted power, on the permeability of the surrounding media, on the alignment of the transmitter-receiver loops, and on the bending of the receiver coil. Systematic measurements demonstrate that the chip is able to deliver a power of about 8 mW and 12.6 mW for 2 W and 12 W transmitted power (P_TR_), respectively, corresponding to a biasing current of both the green and blue LED of 2.8 mA and 4.5 mA (Figure 2C). Such power levels are reached in air when the device is placed parallel to and at 8 cm above the transmitted coil. To simulate realistic biological environments and estimate the impact of the absorption of the RF field in various media, the tests are repeated by surrounding the device with a plastic bag filled with water (Supplementary Figure S5 shows an image of the set-up). In water, the LED turns on only when P_TR_ is greater than 4 W while showing similar output characteristic to the in-air experiment for higher P_TR_ values. Such levels of current correspond to optical power density in the range of 10 mW mm^–2^ for blue and green LEDs and up to 20 mW mm^–2^ for red LEDs (Figure 2D and Supplementary Figure S6) and they are, hence, sufficient for *in vivo* optogenetic control of neuronal activity in nerve fibers (Daou et al., 2016) including their peripheral and spinal axon terminals (Daou et al., 2013). It should also be enough for excitation of superficial dorsal horn neurons (Bonin et al., 2016). For *in vivo* experiments, the biasing current of the LED is influenced by the mechanical deformation of the device coil during implantation and immobilization in the animal. Moreover, in freely moving animals it also depends on the relative angle between the coil and the external magnetic field. Figure 2E shows that the inductive coupling is sufficient to activate the NFC chip even when the device coil is bent down to a 10 mm radius, which represents more bending than is typically required in implants positioned on the dorsal hump of 7-week-old mice. Systematic and repeated mechanical experiments show that the biasing current of the LED is not affected by stretching the interconnections up to 60% (Supplementary Figure S7). The maximum tilting angle between the device and external field is about 50° (Figure 2F), but repeated systematic experiments demonstrated reliable operation across the cage for angles lower than 30°. This aspect constitutes the most severe limitation to a proper reliable functioning of the device since the simplicity of the electronic design does not provide any energy buffer.

The transmitted power and the modulation of the switching of the LED must be set to avoid an increase of the temperature above 1°C. The maximum temperature increase occurs near the location of the NFC chip as opposed to that of the LED. *In vivo* measurements in mice performed by suturing two miniaturized temperature sensors (Negative Temperature Coefficient, NTC) above the chip and the LED and connecting them to an external multimeter (see Supplementary Figure S8 for more details) show that the 1°C limit is achieved for the case of 12 W as transmitted power and for 20% duty cycle (Figure 2G). A comparison between the *in vitro* and *in vivo* tests highlight the beneficial effect of the body temperature homeostasis and blood circulation that effectively limit the temperature rise. Such mechanism would help in disperse the heat, which would avoid an excessive increase of the local temperature (Supplementary Figure S8B). Figure 2H shows the voltage across the LED during the switching operation at 5 Hz, 50 Hz and 500 Hz corresponding to light pulses of 200 ms, 20 ms and 2 ms, respectively. The maximum switching frequency is limited by the set-up time of the NFC chip and it is about 2 kHz.

### Wireless Optogenetic Stimulation of Nociceptive Fibers

To investigate whether wireless optogenetic stimulation can activate nociceptors and elicit nocifensive behaviors, we implanted wireless blue LEDs onto the dorsal surface of the lumbar spinal cord segments L4/L5 in transgenic mice expressing channelrhodopsin 2 (ChR2) in nociceptive sensory neurons (sns-ChR2 mice) (Supplementary Figure S9a) and assessed their behavioral response to blue light stimulation in a place-aversion paradigm (Supplementary Figure S9b). We found that sns-ChR2 mice spent significantly less time in the chamber coupled to the LED stimulation (392 ± 62 s versus 681 ± 40 s, in the stimulation paired versus non-stimulation paired chamber, respectively, *n* = 4 mice), while no significant differences were observed in ChR2-negative control mice which spent on average even more time in the stimulation coupled chamber (592 ± 48 s versus 465 ± 39 s, *n* = 4 mice) (Supplementary Figure S9c). The latter result indicate that a potential increase in local tissue temperature due to LED activation was not strong enough to activate heat-sensitive nociceptors (Mishra et al., 2011).

**FIGURE 3:**
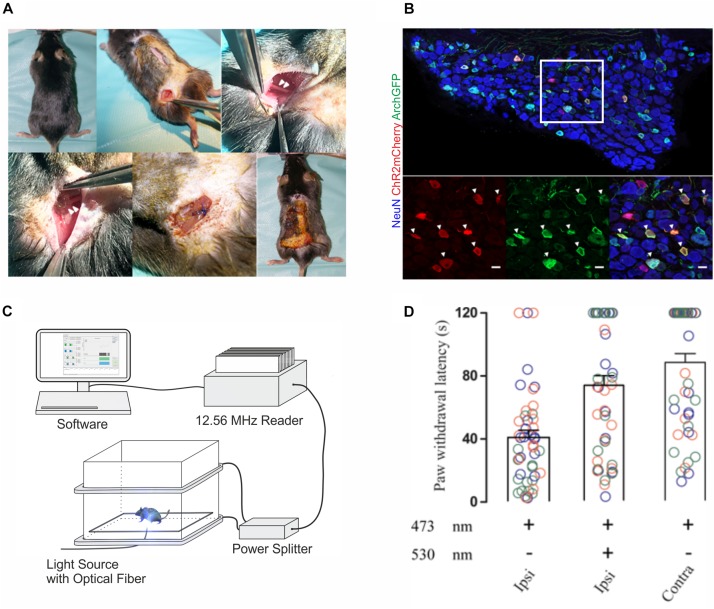
*In vivo* implantations and inhibition of nociceptive afferents with 1-LED device. **(A)** Surgical implantation procedure of single green-LED device at the sciatic nerve; arrowheads on the top panel indicate the sciatic nerve, and on the bottom panel show the placement of the green LED proximal to the sciatic nerve trifurcation. **(B)** Immunohistological stainings of lumbar DRG neurons with antibodies against NeuN, mCherry and GFP. Bottom panels show a higher magnification of the area indicated in white in the top panel. Triple-positive neurons are indicated by white arrow heads (scale bar 20 μm). **(C)** Schematic representation of the behavior experiment measuring the antinociceptive potential of optogenetic inhibition. A mouse was placed on a glass plate between the transmitting coils and restricted in a well-ventilated plexiglas box. ChR2-expressing peripheral afferents were activated with blue light at a wavelength of 473 nm using a fiber-optic light guide coupled to an LED light source, and nerve fibers co-expressing Arch were simultaneously inhibited *in vivo* with green light at a wavelength of 530 nm transmitted from the wireless device at the sciatic nerve. **(D)** Paw withdrawal latencies were recorded upon peripheral blue light stimulation only and a combined peripheral blue light stimulation with green light inhibition at the sciatic nerve on the ipsilateral side, and blue light stimulation only, on the contralateral side. Color codes blue, orange and green indicate recordings on three different animals, and latency measurements were repeated 16 times per stimulation in each animal, as indicated by the number of circles. Statistical significance was calculated with R computational algorithm.

We next investigated whether wireless optogenetics could also be used to suppress nociception. To this end, we tested whether nocifensive behavior elicited by optogenetic ChR2-mediated excitation of peripheral cutaneous nociceptor endings could be counteracted with an optogenetic Archaerhodopsin (Arch)-meditated inhibition of nociceptor axons at the level of the sciatic nerve. To ensure a large overlap of the neuron populations expressing the excitatory ChR2 and the inhibitory opsin Arch, we co-injected two adeno-associated viruses (AAV) that contained expression cassettes for cre-dependent (flexed) ChR2 and archaerhodopsin (Arch) into the sciatic nerve of SNS-cre transgenic mice. Two weeks after AAV injection, a green-light emitting wireless device was implanted proximal to the trifurcation of the ipsilateral sciatic nerve (Figure 3A). Immunohistochemical analysis revealed a large overlap of ChR2 and Arch expression in lumbar DRG neurons (Figure 3B). Paw withdrawal latency measurements were performed 1–2 weeks after AAV injection (Figures 3C,D). Transcutaneous optogenetic activation of nociceptor terminals in the glabrous skin led to robust paw withdrawal reactions with average latencies of 40.9 ± 4.6 s (*n* = 48 trials in 3 mice). We then combined this peripheral transcutaneous nociceptor activation with optogenetic inhibition of action potential propagation along nociceptor axons at the level of the sciatic nerve by a wireless green LED (λ = 530 nm). Under this condition, the latency of the hind paw withdrawal was significantly increased (73.2 ± 6.1 s, *n* = 48 trials in 3 mice; *p* = 0.0001). This reaction time was not significantly different from those observed upon blue light stimulation of the hindpaw contralateral to the virus injection (87.7 ± 5.6 s, *n* = 48 trials in three mice; *p* = 0.15; for details of the statistical analysis see Supplementary Table S1).

### 2-LED Prototype

**FIGURE 4:**
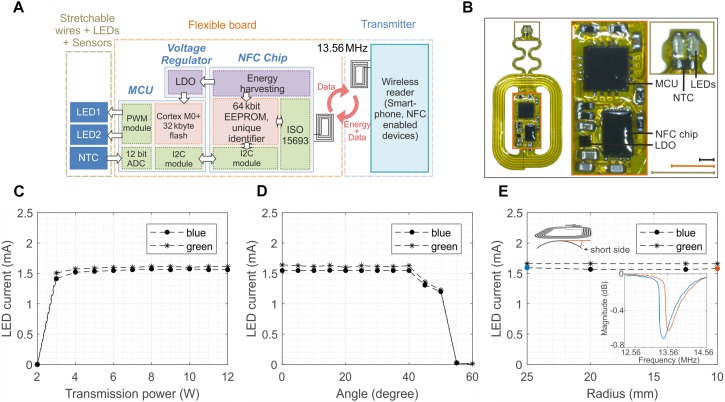
Design and performance of 2-LED device. **(A)** Schematic of the wireless device which incorporates 2 LEDs and 1 temperature sensor (NTC) which are powered via the energy harvesting circuits of the NFC chip, as in the 1-LED version. The addition of a low-drop-out regulator (LDO) ensures stable voltage to a microcontroller (MCU) which is programmed to control the power and the frequency of the LEDs and digitalize the temperature of the NTC. **(B)** Optical image of the wireless device. The device is 29 mm × 15 mm large and weights about 130 mg. The design of the antenna is the same of the one for 1-LED device (scale bars 3 mm for the identical color coded inlets). **(C)** Biasing current of the blue and green LED as a function of the transmitted power. The regulated voltage of the LDO ensures a constant biasing for P_TR_ larger than 4 W. **(D)** Dependence of the current of the LEDs on the tilting angle between the device and external magnetic field. The current and, hence, the LED illumination remain constant for angles smaller than 40°. **(E)** Current of the LEDs when the antenna is bent around various bending radii. The regulated voltage is beneficial to make the current insensitive to the bending despite the shift of the resonant frequency and as long as the magnetic field is sufficiently strong to turn on the NFC.

More intricate experiments, for example targeting the activation of excitatory rhodopsins and the silencing of inhibitory rhodopsins and the simultaneous measurement of important physiological parameters such as temperature, calls for the development of devices carrying multiples LEDs which can be independently activated on-demand, and sensors whose information is transmitted wirelessly in real time. Figure 4 presents a more sophisticated version of the device presented in Figure 1. The prototype implements some of the aforementioned functionalities via the integration of two LEDs and one temperature sensor (NTC) whose selective control and reading require the addition of a microcontroller (MCU, MKL03Z32VFG4 and a Low-Dropout regulator (LDO, TLV70528YFPT) while the communication still relies on the NFC technology (Figure 4A and Supplementary Figure S10). The device can be programed and controlled via a custom-made software, including LED brightness in 11 steps from 0 to 100% and modulation patterns with a resolution from milliseconds to seconds (see Supplementary Figure S11 for an example of the graphic interface). It stores the configuration during a power loss and can be reconfigured in real-time. The construction and the layout of the device are similar to the one described previously (Figure 4B). The advanced electronic design ensures more robust operations compared to its 1-LED counterpart. In fact, the biasing current of the LEDs and, hence, its optical power are almost insensitive to the transmitted power (Figure 4C), the tilting angle (Figure 4D) and the level of bending (Figure 4E). Supplementary Videos S1, S2 provides examples of the devices during operation *in-vivo* (implanted in a mouse) and *in-vitro* respectively. Mechanical stretching of the interconnections showed no significant variation of the current of the LEDs for strain up to 30% (Supplementary Figure S12). Such behavior is mostly due to the regulated output of the LDO, which power the LEDs and the NTC. It is worth mentioning that this new design simplifies the power set-up since the external magnetic field does not need to be pulse modulated since the MCU is responsible for the switching of the LEDs.

**FIGURE 5:**
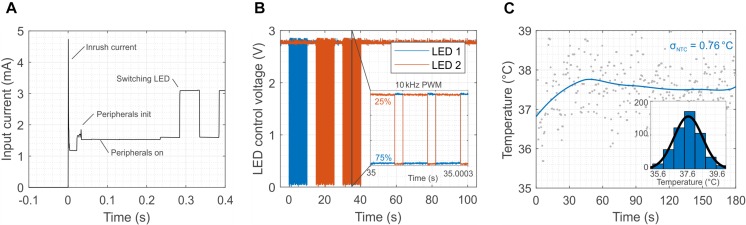
Initialization, switching and *in vivo* temperature measurement for the 2-LED device. **(A)** Initialization phase of the device. The plot highlights the time required for the set-up of the peripherals (300 ms) and the power sharing between the MCU and the LEDs. The MCU consumes about half of the available power (1.5 mA). **(B)** The switching of the LEDs is controlled by the micro-controller, and it can reach a frequency as high as 10 kHz, which is sufficient for optogenetics. Moreover, the MCU allows for individually controlling the power of the LEDs through the duty cycle of the modulation of the output. **(C)** Calibrated temperature measurement close to the LEDs after switching them on. The coupling of the external magnetic field in the analog domain of the device causes a standard deviation of 0.76°C in idle state.

Such sophisticated and robust operations come at the price of higher power consumption, especially of the microcontroller. Figure 5A shows the power consumption of each component of the system during the initialization phase (when the external field is switched on). The MCU consumes almost half of the power available (1.5 mA) and it has a delay of about 0.3 s before it activates the LEDs. However, after the initialization, the MCU is able to switch the LEDs at frequency as high as 10 kHz (Figure 5B) against the 2 kHz of the first prototype (Figure 2H) with a programed duty cycle, which allows for tuning the illumination power independently from the transmitted external field. Another salient feature of the design is the possibility to wirelessly monitor in real time the temperature near the LEDs. Potential tissue heating is a two-fold concern. First, thermal tissue damage occurs at temperatures above 43°C (Yarmolenko et al., 2011). Second, temperatures above 43°C activate TRPV1 channels (Tominaga et al., 1998) that function as detectors of noxious heat. Their activation elicits (heat) pain sensations and triggers nocifensive behavioral reactions. Figure 5C shows the temperature during a typical *in vivo* optogenetic experiment. The reading of the sensor, after the application of a low-pass filter, exhibits a temperature increase smaller than 1°C with a standard deviation of about 0.76°C. Supplementary Figure S13 also provides a comparison between the external NTC placed in the vicinity of the LEDs and the temperature sensor embedded in the micro-controller. The larger standard deviation registered in the external NTC is probably caused by the coupling of the interconnections with the large external magnetic field. The internal temperature sensors of the micro-controller, however, do not provide accurate information on the temperature in the proximity of the LEDs but rather on the temperature of the chip. From Supplementary Figure S13 it is obvious that the variance of the measurement set a limit to the response time. As shown in Supplementary Figure S14, averaging the measurement over 30 s intervals reduces the deviation to ± 0.34°C, which we consider an appropriate accuracy for typical *in vivo* experiments (± 1°C considering 3 times the standard deviation). Provided that a large enough safety margin is applied (e.g., 40°C, or 3 K less than the thermal tissue damage threshold of 43(°C), the resulting delay of 30 s appears acceptable.

## Discussion

Nociceptors are peripheral nerve cells specialized to detect stimuli that threaten tissue integrity. They convey action potentials generated in their peripheral endings to the central nervous system. The majority of their central projections terminate in the superficial dorsal horn of the spinal cord (Braz et al., 2014; Usoskin et al., 2015). Nociceptors are therefore essential for the generation of protective responses to acute painful stimulation. Their activity is believed to also critically contribute to the generation and maintenance of chronic pain syndromes. Effective control of their activity is therefore a promising approach to chronic pain treatment. Optogenetic control may constitute a particularly appealing approach to nociceptor control as it allows extremely precise temporal control with onset and offset times in the millisecond range.

In the present report, we describe a fully wireless device for optogenetic experiments. Its small size and low weight enable implantation in mice on top of the spinal cord or in close proximity to a peripheral nerve without causing any obvious impairment. Such devices are highly desirable in all experiments that benefit from unrestrained mobility. The majority of behavioral optogenetic experiments are done with implanted fiber optics connected to a peripheral stationary light source such as a laser or an LED. Such devices have several disadvantages. Experiments involving complex environments containing tunnels, labyrinths, nests, houses or other elements of so-called enriched environment are basically incompatible with devices that require implanted fiber optics. They are also highly problematic when more than one freely behaving mouse is to be tested in the same experimental compartment. It is, however, well-established that mice behave differently and exhibit probably more physiological behavior when examined in a more complex environment (Kempermann et al., 2002; Freund et al., 2013; Körholz et al., 2018). Similarly, mice are highly social animals and their behavioral repertoire is strongly impaired if the mice are deprived from social interactions (Valzelli, 1985; Matsumoto et al., 2005; Fone and Porkess, 2008). Many tests in behavioral and psychiatric research actually investigate the interaction between individuals (Koolhaas et al., 2013; Pasciuto et al., 2015; Chang et al., 2017). In all these cases, optogenetic experiments will largely benefit from fully wireless technology or may even only become possible with such devices. It should also be noted that animal legislations require that single housing of mice is reduced to the absolute required minimum. In case of optogenetic experiments requiring fiber optics connected to implanted cannulas that stick out through the skin of the mouse, such single housing can be absolutely necessary to avoid injuries and damage to implanted cannulas by cage mates. The fully wireless devices described here avoid these problems and are also preferable from an animal welfare perspective. A potential problem of the wireless devices described in this report is that their powering may vary with the position of the mice in the arena or the posture of the mice. Our place aversion experiments indicate that, although such fluctuations occur, they do not compromise the experiment to a relevant extent. It is worth mentioning that the system is built around the ISO 15693 “vicinity cards” standard with a typical reading range of 20 cm. For security reason, the underlying RFID technology is engineered for short-range data communication rather than for energy transfer. For larger ranges a technology as that from Powercast could be applied^1^. More effective solutions to stabilize the power delivery consist in integrating buffer energy storage components. First, a supercapacitor, like the Seiko Instruments CPH3225A series capacitor (3.20 mm × 2.5 0 mm × 0.90 mm) with 11 mF the device could overcome an RF power transmission loss. A second option is a rechargeable battery in combination with a battery managing circuit, which would increase significantly size and weight of the implant and thus complicates an *in vivo* application in mice. A battery such as the GMB GMB031009 (10 mm × 9 mm × 3 mm) with 12 mAh would supply the device autonomous for over 4 h.

While optogenetics have become highly instrumental in experimental systems neuroscience (Deisseroth, 2015), their translation to patient therapy including pain therapy will likely face challenges (Beaudry et al., 2017). Considerable progress has been made in the development of miniaturized and biocompatible devices for light delivery. Similarly successful were attempts to reduce phototoxicity through the development of red-shifted effector opsins that allow the use of red or even near-infrared light for stimulation (Lin et al., 2013; Chuong et al., 2014; Mager et al., 2018). While blue light causes considerable phototoxicity, this effect is marginal with red and near-infrared light (Mager et al., 2018). Other challenges that are related to transgene expression of the effector protein still pose significant hurdles. In mice, cell type-specific expression can be achieved via the cre-loxP or similar system. In humans, cell type-specific expression will depend on the identification of specific promoter sequences [“minipromoters” (de Leeuw et al., 2016; Portales-Casamar et al., 2010)] short enough to be compatible with the limited packaging capacity of viral vectors. For some applications, especially in the peripheral nervous system, neuron subtype-selectivity may be less important and the development and choice of appropriate serotypes may suffice (Iyer et al., 2014). When repeated AAV injections are needed (e.g., because of transgene inactivation), the generation of inactivating antibodies can pose important limitations. Potentially even more problematic is the immunogenicity of the optogenetic proteins that may provoke cytotoxic immune reactions (Maimon et al., 2018) that would be particularly devastating in post-mitotic neurons. However, in the light of the steady progress in gene therapy (Galvan et al., 2017) we are cautiously optimistic that these challenges will be mastered within reasonable time frames.

We are convinced that our wireless technology has potential for applications beyond experimental neuroscience. Our experiments have shown that inhibitory opsins can be used to block nociceptive signal propagation at the level of a peripheral nerve, in our case the sciatic nerve. Such inhibition may be desired therapeutically in patients who suffer from localized pain syndromes or mononeuropathies. The ability to precisely control the activity of our wireless devices transcutaneously with small portable electronic devices such as mobile phones would be ideal for fast on-demand therapeutic intervention. This may be particularly relevant in cases of so-called break-through pain attacks (Patt and Ellison, 1998) that are frequently observed in cancer pain patients even when these patients receive adequate analgesic therapy with opioid analgesics. Blockade of incoming nociceptive signals at the level of the spinal cord or dorsal roots may be highly desirable in patients with radicular pain syndromes, for example those caused by lumbar disc hernia.

## Materials and Methods

### Assembly of the Devices

Preliminary prototypes of the devices were built in a cleanroom environment by following conventional lithography, etching, and vacuum deposition steps, while a frozen-design version was realized with the help of a Colorsunny Electronic^2^ by laser cutting technologies. The in-house fabrication process involved the following steps:

1. A Cu foil (18 μm thick, Oak Mitsui Micro-thin series) provided the material for the coil. A film of polyimide (PI; 25 μm thick, PI2545, HD Microsystems) spin-cast 10 times onto the Cu foil at 2000 rpm for 30 s, baked on a hot plate at 150°C for 5 min, and in a vacuum oven at 250°C for 75 min formed an insulating layer coating. Laminating this PI-coated Cu foil onto a PDMS (10:1, Sylgard 184) coated glass slide, with the PI side down, allowed for patterning of the Cu foil into a coil geometry by photolithography and wet etching (CE−100 copper etchant, Transense, ∼2 min with frequent rinsing by water). Another PI spin−cast film formed by following the aforementioned procedure covered the coil. Photolithography (AZ 4620) and oxygen plasma etching (200 m Torr, 20 sccm O_2_, 150 W for 900 s) created via holes through the PI. Oxide remover (Flux, Worthington) eliminated the copper oxide on the pads exposed at the base regions of the via holes. Electron beam evaporation of Cu (18 μm thick) followed by photolithography (AZ 4620) and wet etching (copper etchant) defined a pattern of interconnects. Spin casting yielded another 25 μm thick layer of PI over the entire patterns. Electron beam evaporation of a 50 nm thick layer of SiO_2_ followed by photolithography (AZ 4620) and reactive-ion etching created a hard mask for removal of the PI by oxygen plasma etching in all regions except for those above the traces for the coil and interconnects.

The components are glued on the flexible PCB via a low temperature epoxy glue (EPO-TEK^®^ H20E - Epoxy Technology).

The 1-LED devices contain the following components:

**Table:** 

NFC chip	M24LR64E-RMC6T/2		
C1	0402, 50 V	160 pF	± 2%
C2	0402, 50 V	14 pF	± 2%
R1	0402, 0.063 W	49.9 Ω	1%
LED1	LT QH9G-Q200-25-2Z4Y, 0402, green 530 nm		

The 2-LED devices contain the following components:

**Table:** 

Regulator	LDO, TLV70528YFPT, 2.8 V, 200 mA,		
NFC chip	M24LR64E-RMC6T/2		
Microcontroller	MKL03Z32VFG4		
C1	0402, 50 V	160 pF	± 2%
C2	0402, 50 V	14 pF	± 2%
C3	0402, 6.3 V	1 μF	± 10%
C4, C5	0402, 6.3 V	100 nF	± 10%
R1, R2	0402, 0.063 W	75 Ω	1%
R3	0402, 0.063 W	100 kΩ	1%
R4, R5	0402, 0.063 W	10 kΩ	1%
R6	0402, 0.063 W	54.9 kΩ	1%
R7	0402, 0.063 W	0 Ω	
PT1	NTC, ERT-J0EV104G, 0402	100 kΩ	2%
LED1	LB QH9G-N100-35-1, 0402, blue 470 nm		
LED2	LT QH9G-Q200-25-2Z4Y, 0402, green 530 nm		
			

After mounting the components onto the flexible PCB, small drops of transparent epoxy are used to encapsulate the chips. Last consists in covering the entire system with a thin layer of PDMS which is deposited by spin coating.

### Mechanical Simulations

The commercial software package ABAQUS allowed for simulating the mechanical response of interconnection of the device between the antenna and the LED. The composite layer (PDMS, Cu, and PI) consisted of a 6-node linear triangular prism (C3D6). The simulations implemented values of the elastic modulus of PDMS, PI, and Cu of 2, 2000, and 127,000 MPa, respectively (Musto et al., 2004; Xu et al., 2011; Wang et al., 2014). The simulations also implemented linear elastic constitutive models for each constituent but included non-linear geometric effects (finite deformation) to enable large out-of-plane deformation. Additionally, to find when plastic deformation occurred in the composite, a yield strength of Cu of 80 MPa was considered as a representative value (Zhang et al., 2007).

### Transmitted Coils and Electronics

The transmitted coils (ID ISC.ANT310/310) have been bought from FEIG Electronic, arranged in Helmholtz configuration and retuned at 13.56 MHz with the help of a Network Analyzer (HP 8753E). A power splitter (FEIG ID ISC.ANT.PS) was also used to connect the coils to the power source. The power source consisted of a signal generator (Keysigh 33522A), a power amplifier (Mini Circuits ZHL-100W-52X-S +), a laboratory power supply (Elektro-Automatik EA-PS 3032-10B), and an attenuator (Mini Circuits BW-N3W20 +).

### Thermoelectric Characterization of the Devices

#### Measurement of Power Distribution in the Helmholtz Coil Set-Up

The electrical field distribution and thus the special influence on the wirelessly transmitted electrical power was measured in the active chamber in a single coil and Helmholtz coil configuration (Figure 2B). To measure the power a Power Meter (Anritsu ML2437A) with a Power Sensor (Anritsu MA2481D) was connected to our matched rectangular loop antenna. During the measurements the loop antenna was placed parallel to the transmitter coils.

#### Output Power of the Devices and Its Dependence on Surrounding Media, Bending, and Tilting

The electrical response of the device to the wirelessly transmitted power was measured in various conditions (Figures 2C,E,F) and by placing the device in the middle of the cage. The biasing current of the LED was measured via wired connections to a Digital Multimeter (Keysight 34465A). It is worth mentioning that the connecting wires were carefully twisted to minimize the coupling with the high external field.

Measurement in water (Figure 2C): a standard plastic bag was filled with water and wrapped around the device, which was positioned in the middle of the cage. The total thickness of the water surrounding the device was about 2 cm (1 cm from the top and 1 cm from the bottom).

Bending tests: the device was bent around a plastic rod of various radii and the current of the LED was measured with a multimeter. The shift of the resonant frequency (inset Figure 2E) during bending was captured with a Network Analyzer (HP 8753E).

Tilting tests (Figure 2F): the device was placed on a plastic arm whose angle with respect to the external field was adjusted with a 5° step. The current of the LED was measured with the multimeter.

#### Optical Response of the LEDs

The output power levels of wired LED devices (Figure 2D) were measured with a Digital Handheld Optical Power Meter (ThorLabs PM100D) while biasing the devices with various currents from a Source/Measurement Unit (Keysight B2902).

#### Thermal Behavior of the Devices and LEDs

The increase of the temperature during wireless operation of the devices was measured by connecting two miniaturized NTCs (Panasonic ERT-J0EV104G) to a Precision Source/Measurement Unit (Keysight B2902A). The NTCs were positioned on top of the LED and of the NFC chip with the help of non-conductive transparent epoxy glue.

#### Switching Frequency

The coupling between the modulation of the transmission field and the LED was measured on the energy harvesting output of the NFC chip with an Oscilloscope (Keysight DSOX3054T). It is worth mentioning that despite twisted wires and shielded measurement probes the carrier frequency of the wireless transmission is present in the acquired data.

### 2-LED Devices

#### Set-Up for *in vivo* Measurement of the Temperature

The experiments involving the wireless real-time reading of the temperature with the 2-LED devices were carried out with a FEIG electronic (ISO15693 LONG RANGE READER MODULE, ID ISC.LRM2500) wireless reader. The reader is able to provide 12 W output power.

#### Software for *in vivo* Measurement of the Temperature

The PC application is used to interface the wireless reader and to configure the 2-LED devices. It is built on the FEIG function library V4.07.00 with a custom-made user interface written in Python 3 using the PyQt framework. Its modular implementation allows to flexibly adapt the interface to different implantable devices.

If a 2-LED device is in the range of the reader, the software will detect the implant and allows to individually reprogram the LED brightness by changing the PWM duty cycle as well as the LED on and off time. The software periodically reads the configuration of the individual devices to ensure proper configuration. Furthermore, programmable overlaying patterns allow for defining specific repeatable experiments. In the case of multiple devices in the reader range, devices can be addressed and configured individually. During experiments, the die temperature of the microcontroller as well as the temperature in the vicinity of the LEDs is visualized in real-time and logged in Matlab and Microsoft Excel compatible file formats.

### Animals and Adeno-Associated Viruses (AAVs)

AAV.CAG.flex.hChR2(H134R)-mCherry and AAV.CAG.flex. Arch-GFP (Penn Vector Core) were mixed in equal titers and used for co-injections into the left sciatic nerve. SNS-Cre mice (Tg(Scn10a-cre)1Rkun) (Agarwal et al., 2004) were used for virus injections and sciatic nerve implantations. These mice were bred with Ai32 ChR2 reporter mice (R26^LSL–ChR2–YFP^) to generate SNS-ChR2 transgenic mice for spinal cord device implantations. All animal experiments have been approved by the veterinary office of the canton of Zurich (license number 174/2016).

### Virus Injections and Wireless Device Implantations

#### Intraneural AAV Injections

Adeno-associated virus (AAV) injections were performed in 6–7 week-old-male mice. Mice were anesthetized under 2–5% isoflurane and maintained under 1–2% isoflurane until completion of the surgical procedure. Buprenorphine (0.003 mg/ml) was administered subcutaneously before the start of the surgery for optimal intra- and post-operative analgesia. The dorsal skin was shaved and disinfected with 1% betadine solution. A small incision was made lateral to the midline, and the skin was retracted to expose the gluteus superficialis and the biceps femoris muscles. The connective tissue was dissected with blunt forceps to expose the sciatic nerve. The nerve was gently lifted above of the cavity using blunt forceps. AAV suspensions were injected into the sciatic nerve in a volume of 3 μl over 3 min using a Hamilton syringe hand-held and maintained at an angle of 45° for steady infusion of the liquid into the nerve. The syringe was removed ∼1 min post-infusion of the virus and the muscles were sutured with absorbable sutures (Safil 4-0, B. Braun). The skin was sutured with non-absorbable sutures (Dafilon 5-0, B. Braun) and the mouse was allowed to recover on a heat pad. After surgery, all mice were group-housed in their home cages.

#### Sciatic Nerve Implants

Three weeks after the virus injections at the sciatic nerve, the mice were implanted with the wireless green-LED. The mice were anesthetized and maintained under 1–2% isoflurane until completion of the surgical procedure. Buprenorphine (0.003 mg/ml) was again administered subcutaneously before the start of the surgery. An incision was made along the mediolateral axis at the ipsilateral dorsal posterior end. The gluteus superficialis and the biceps femoris muscles were dissected and retracted yet again to expose the sciatic nerve in the cavity. The LED atop the wireless device was carefully extended by stretching the copper wire and placed superficially over the sciatic nerve, proximal to the trifurcation of the common peroneal, tibial, and sural nerves. The muscles were sutured with non-absorbable sutures to minimize movement of the LED within the cavity. The bulk of the device was placed over the dorsal hump and loosely sutured on parallel sides to prevent post-surgery movement and associated discomfort for the mouse. The skin was sutured and the mouse was allowed to recover on a heat pad. After surgery, all mice were group-housed in their home cages.

#### Spinal Cord Implants

Six- to seven-week-old male mice were implanted with wireless blue-LEDs on the dorsal surface of the lumbar spinal cord segments L4 and L5. Anesthesia was induced using 2–5% isoflurane and maintained at 1–2% isoflurane until completion of the surgery. Buprenorphine (0.003 mg/ml) was administered subcutaneously before the start of the surgery. The dorsal skin was shaved and disinfected with 1% betadine solution. An incision was made along the midline, and the skin was retracted to expose the dorsal hump and the vertebral column. Implantation of the LED was targeted at the L4-L5 spinal cord segment; therefore, laminectomy of the T13 vertebral disc was performed prior to implantation. Incisions were made on the muscles lateral to the tendons spanning either sides, and the vertebral column was clamped with spinal adaptors. The T13 vertebral disc was exposed, and the tissues covering the spinous and transverse processes of the disc were removed using forceps. The processes were then removed using a bone trimmer. The dura mater spanning the L4-L5 segments was exposed, and collagen strips (Lyostypt, B. Braun) were used to control bleeding from the muscles. The spinal adaptors were removed and the wireless device was placed with the LED superficially over the L4-L5 spinal segment. The copper wire carrying the LED was sutured with the muscles lateral to the tendons on both sides. In addition, a small amount of tissue glue was applied to seal the muscles over the LED, as to prevent its movement. The skin was sutured over the device. Mice were allowed to recover on a heat pad and were then transferred to their home cages with food and water *ad libitum*.

The LEDs typically had a distance from the dorsal spinal cord surface of about 100 μm (measured after completion of the experiments with a software-controlled micromanipulator). The scattering during penetration through the spinal cord white and gray matter tissue has been shown to decreases the power of the excitation light by 65–75% at a depth of 250 μm (Samineni et al., 2017). This depth corresponds to the border between lamina II and III. Since sensory including nociceptive fibers run in the white matter on top of the grey matter, we estimated should be exposed to between 10 mW/mm2 (at the spinal cord surface) and 2.5 mW/mm2 (at the boarder between laminae ii and III).

#### *In vivo* Behavior Experiments

Mice were used for behavior experiments 48 h after LED implantation at the sciatic nerve and 1 week after implantation at the spinal cord. Mice were placed on a well-ventilated Plexiglas box with a glass base within the powering coils and ambient noise was created by turning on the fan for the amplifier. Mice were allowed to habituate to the experimental setup prior to experiments. The experimenter was blinded to the genotypes.

#### Optogenetic Inhibition of Nociceptive Fibers

For optogenetic inhibition of nociceptive fibers, withdrawal latencies were measured for the ipsilateral and contralateral paw in response to peripheral blue light (λ = 473 nm) illumination via a fiber-coupled LED light source (Thorlabs, Inc.) at an output power of 3.0 mW/mm^2^ through a glass base. The experimental cut off was set at 120 s. To test the responses of ChR2 excitation at periphery and simultaneous inhibition of Arch at the sciatic nerve level, withdrawal latencies were measured on the ipsilateral paw to blue light illumination and simultaneous green light illumination (λ = 530 nm) at the sciatic nerve with the wireless LED device on the continuous mode at 12 W output power. Repeated measurements were taken and respective averages were calculated.

#### Optogenetic Excitation of Nociceptive Fibers and Place Aversion

For real-time place aversion experiments with spinal cord implants in SNS-ChR2 mice, the animals were habituated in the two chambers place aversion box (Cunningham et al., 2006) for 15 min to allow exploration. The animals were transferred to their home cage until the beginning of the experiment. The powering coils were tuned to have the output power of the devices at 6 W on continuous mode, and the animals were introduced to the box through the central hallway and allowed to freely move between the two chambers, one of which was coupled to the LED stimulation. The aversive behavior was quantified by comparing the average time spent in each of the chambers over a total period of 20 min.

### Immunohistochemistry and Image Analysis

Following the behavioral experiments, mice were deeply anesthetized and transcardially perfused with 4% parafor- maldehyde (PFA) solution in 0.1M phosphate buffer pH 7.4, and the vertebral column was post-fixed in 4% PFA for 2 hrs. The vertebral column was incubated in 30% sucrose cryoprotectant solution at 4°C overnight. L4, L5, and L6 DRGs with roots attached to the sciatic nerve were dissected and embedded in Neg-50 (Thermo Fisher Scientific) frozen section medium. DRG cryosections of 16 (μm were prepared with a Horax C60 Cryostat and mounted on superfrost plus glass slides. The slides were briefly washed with PBS and incubated with primary antibodies (Molecular Probes: rat anti-mCherry, rabbit anti-GFP, and guinea pig anti-NeuN) in 5% donkey serum at 4°C overnight. The slides were rinsed and incubated with respective Alexa-Fluor or Cyanine-dye conjugated secondary antibodies for 1 hr at room temperature. The slides were further washed and mounted with coverslips using DAKO fluorescent mounting medium. The sections were visualized in a Zeiss LSM 800 with Airyscan confocal microscope and z-stacks of fluorescent images were taken using either 10x Plan-Apochromat or 25x Plan-Neofluar oil-immersion objective. The images were acquired and processed using the ZEN blue-edition software.

### Statistics

Repeated measures ANOVA was used for statistical analysis of the place aversion experiments with a *post hoc* Bonferroni correction, and nested ANOVA was applied for calculating significance between the paw withdrawal latencies using the R computational algorithm.

## Data Availability

All data needed to evaluate the conclusions in the paper are present in the paper and Supplementary Materials. Any additional data sets, videos, analysis details, and material recipes are available upon request.

## Ethics Statement

This study was carried out in accordance with the recommendations of “Policy der Universität Zürich zur tierexperimentellen Forschung (www.uzh.ch/cmsssl/en/research/ethics/animalwelfare), Prorektorat Medizin und Naturwissenschaften.” The protocol was approved by the “Tierversuchskommission des Kantons Zürich.”

## Author Contributions

PM, NS, GS, and HZ conceived the study, designed the experiments, analyzed the results, prepared the figures, and wrote the manuscript. GS, SL, and MaP designed the 1-LED device. PM, GS, MV, AM, SL, and MaPh electrically and mechanically characterized the 1-LED device. PM, MiP, MM, and GS designed and characterized the 2-LED device. SL and MaP ran the mechanical simulation to optimize the design. GS, AM, AS, and PM designed the wireless power transmitter and the receiver coils. NS and HJ took care of the surgery, implantation, and the *in vivo* experiments. NS and HZ designed the *in vivo* experiments and analyzed the results. GS, HZ, and GT supervised the whole work.

## Conflict of Interest Statement

GS was employed by company Salvatore Optopharma. AS was employed by company Sensor ID. The remaining authors declare that the research was conducted in the absence of any commercial or financial relationships that could be construed as a potential conflict of interest.

## Footnotes

1 https://www.powercastco.com/

2 http://www.darlox.com/about/about48.html

## Supplementary Material

FIGURE S1 Mask design of the 1-LED device and list of the components.

FIGURE S2 Additional images of the 1-LED device.

FIGURE S3 Schematic of the wireless set-up.

FIGURE S4 Mapping of the power in the xy plane in case of 1-coil and 2-coil in Helmot configuration at different distance from the loop.

FIGURE S5 Set-up for the measurement of the induced current in the LED as function of the transmitted power when the device is surrounded by water.

FIGURE S6 Electrical and optical characterization of the LEDs used in the experiments.

FIGURE S7 Current of the LED when the interconnection is stretched up to 60%. No visible change is observed.

FIGURE S8 *In vitro* and *in vivo* measurement of the increase of the temperature above the NFC chip for various operation conditions of the device.

FIGURE S9 *In vivo* spinal implantation of wireless device and place aversion behavior.

FIGURE S10 Mask design of the 2-LED device and list of the components.

FIGURE S11 PC application user interface designed to configure the wireless reader as well as to program and monitor the 2-LED devices.

FIGURE S12 Current of the blue and green LEDs when the interconnection is stretched up to 30%. No visible change is observed.

FIGURE S13 *In vivo* measured and wireless transmitted temperature. **(a)** Calibrated temperature measurement close to the LEDs after switching them on. The coupling of the external magnetic field in the analog domain of the device causes a standard deviation of 0.76°C in idle state. **(b)** Temperature measured at the microcontroller die.

FIGURE S14 Low pass filtering of wireless temperature measurement. The filtering is applied through an averaging over a moving window with various depth. The plots show the impact of the depth of the window on the standard deviation and on the response time. By increasing the depth the standard deviation decreases and the response time increases.

TABLE S1 Statistical Significance table from R.

VIDEO S1 wireless_test_2LED_ETH, testing of the wireless power transmission.

VIDEO S2 mouse_2LED_ETH, video of an *in vivo* experiments with the 2-LED device implanted.
