# Supplementary material for: Flexible and Lightweight Devices for Wireless Multi-Color Optogenetic Experiments Controllable via Commercial Cell Phones
Source: Front Neurosci. 2019 Sep 6;13:819. doi: 10.3389/fnins.2019.00819 (PMC6743353; doi:10.3389/fnins.2019.00819)
Supplement: FIGURE S1 — Mask design of the 1-LED device and list of the components. [file Data_Sheet_1.pdf]

Supplementary Information

Flexible and lightweight devices for wireless multi-color optogenetic experiments controllable via commercial cell phones

Philipp Mayer, Nandhini Sivakumar, Michael Pritz, Matjia Varga, Andreas Mehmann,

Seunghyun Lee, Alfredo Salvatore, Michele Magno, Matt Pharr, Gerhard Troester,

Helge C. Johannssen, Hanns Ulrich Zeilhofer, Giovanni Antonio Salvatore

E-mail: gasalvatore1982@gmail.com, *zeilhofer@pharma.uzh.ch*

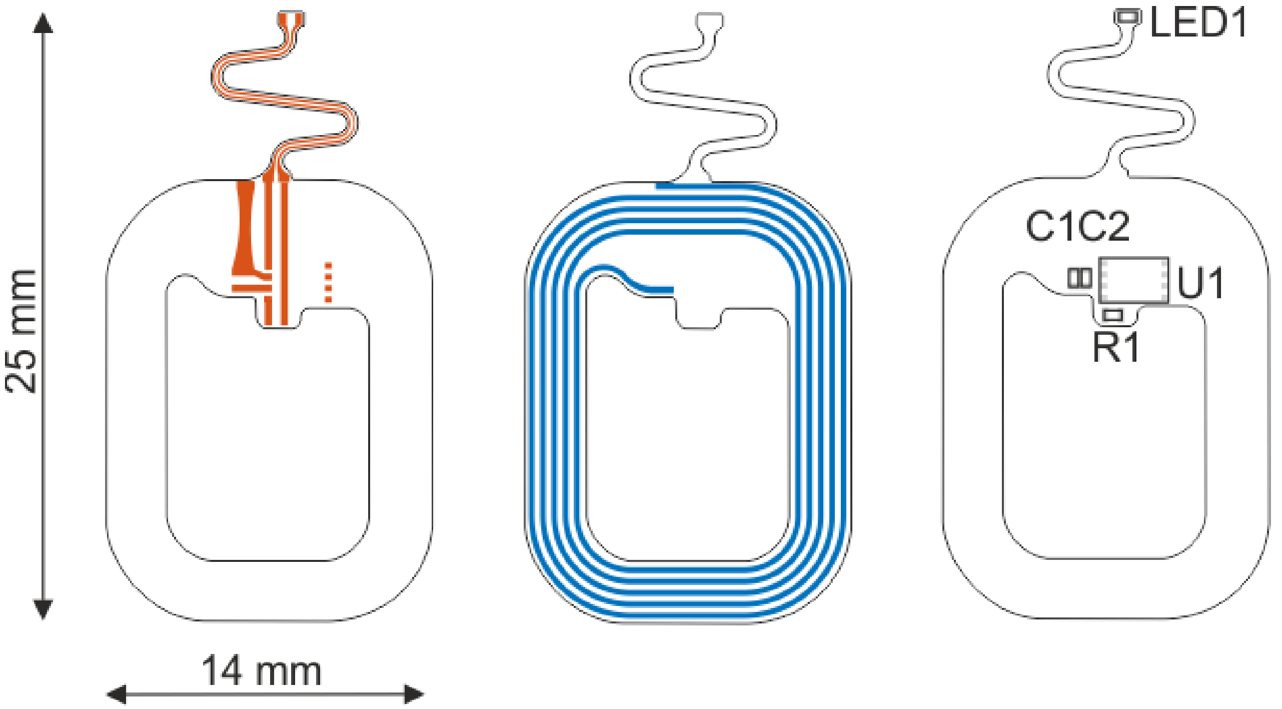

U1	M24LR64E-RMC6T/2		
C1	0402, 50 V	160 pF	±2 %
C2	0402, 50 V	14 pF	±2 %
R1	0402, 0.063 W	49.9 Ω	1 %
LED1	LT QH9G-Q200-25-2Z4Y, 0402, green 530 nm		

Fig. S1 Mask design of the 1-LED device and list of the components.

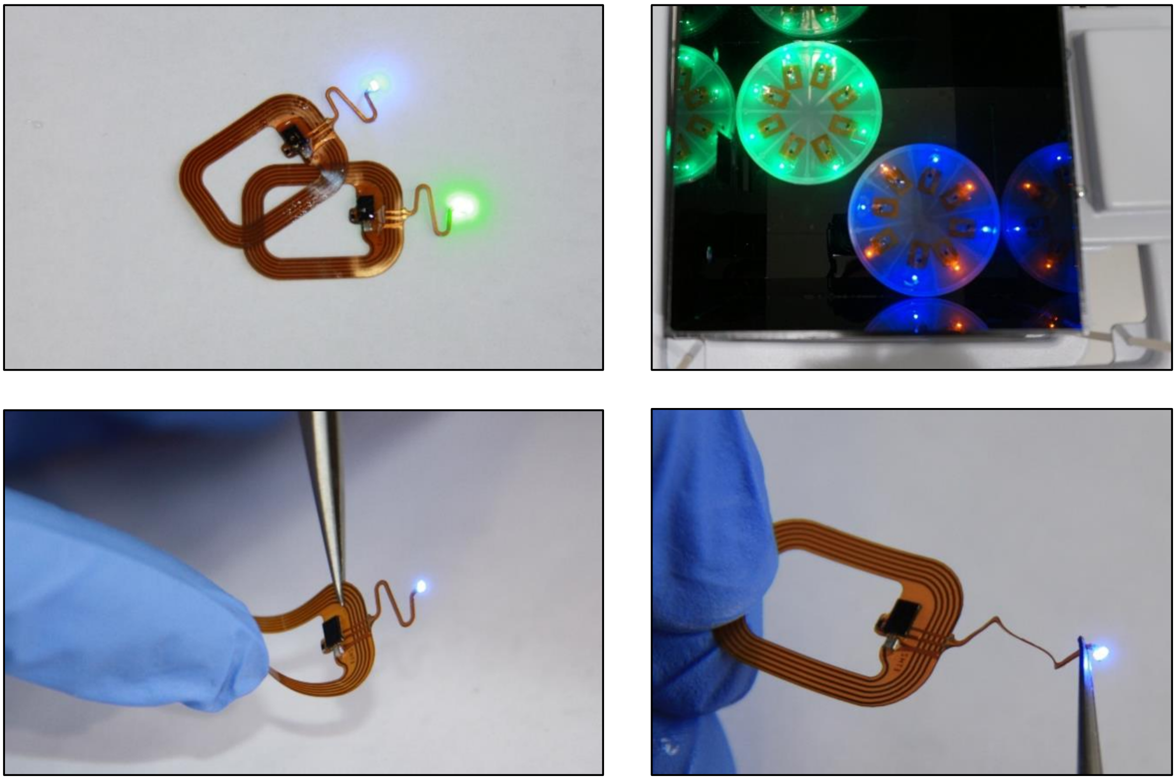

Fig.S2 Additional images of the 1-LED device.

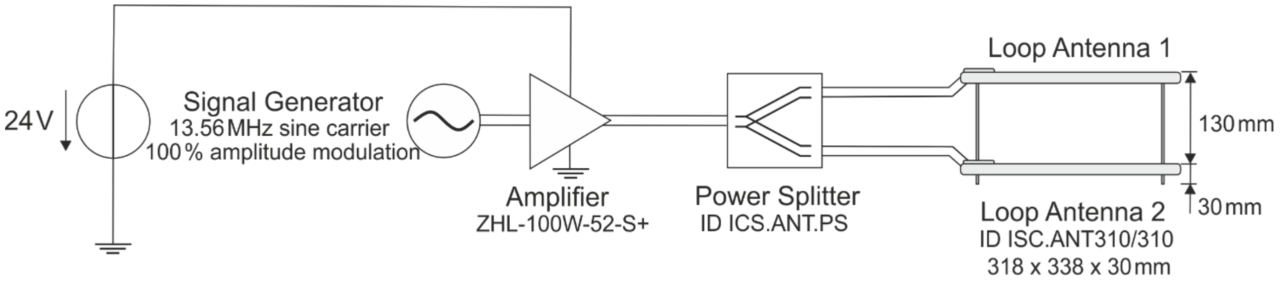

Fig.S3 Schematic of the wireless set-up.

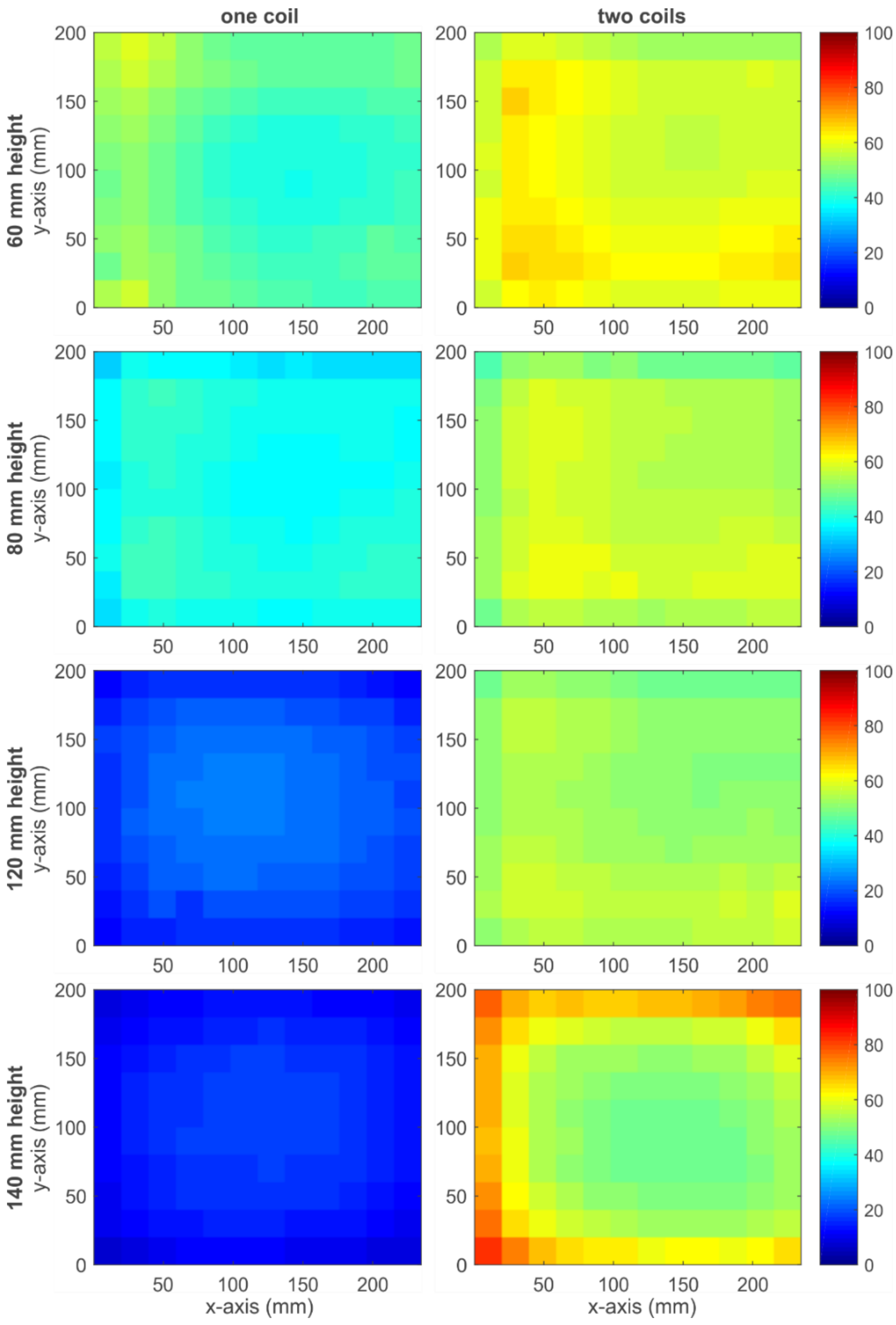

Fig.S4 Mapping of the power in the xy plane in case of 1-coil and 2-coil in Helmotz configuration at different distance from the loop.

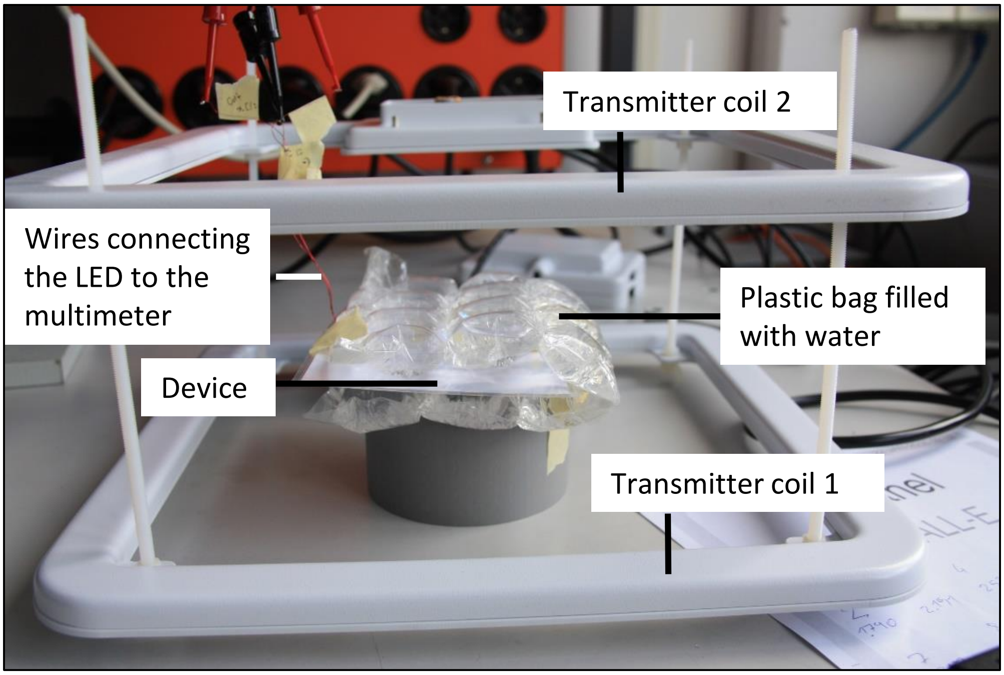

Fig.S5 Set-up for the measurement of the induced current in the LED as function of the transmitted power when the device is surrounded by water.

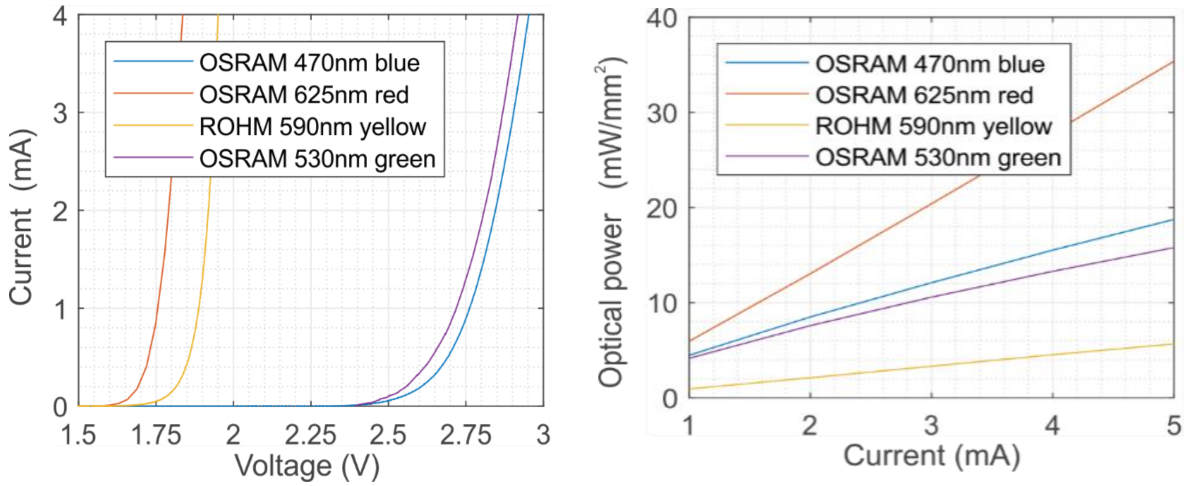

Fig.S6 Electrical and optical characterization of the LEDs used in the experiments.

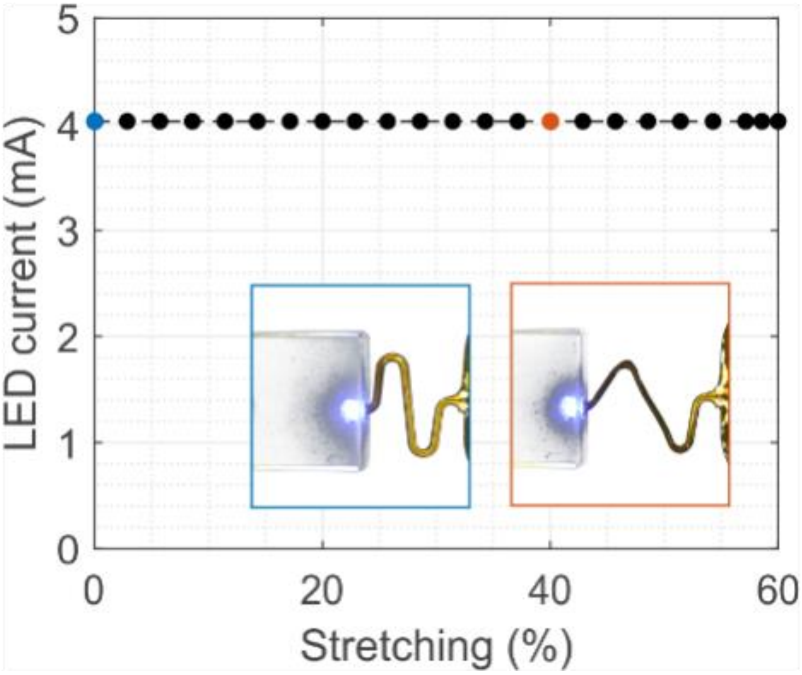

Fig.S7 Current of the LED when the interconnection is stretched up to 60 %.

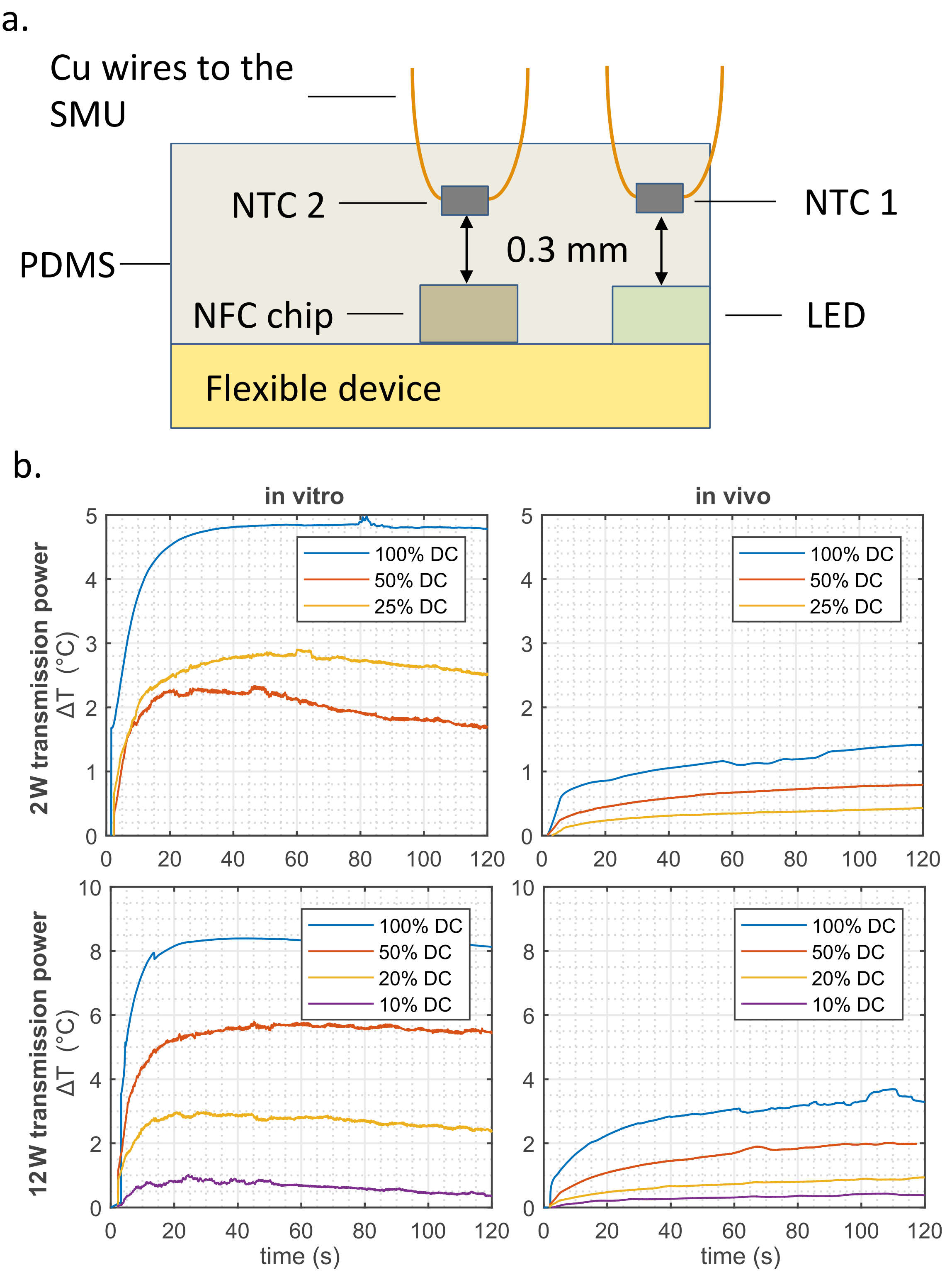

Fig.S8 *In vitro* and *in vivo* measurement of the increase of the temperature above the NFC chip for various operation conditions of the device. a. Drawing of the arrangement of the NTC onto the LED and the MCU. The measurement is performed by connecting the NTC to the Source/Measurement Unit via Cu wires which are twisted to minimize the coupling with the external magnetic field; **b.** Results of the *in vitro* and *in vivo* measurement of the temperature increase for various transmitted power and duty cycles. In-vivo, temperature homeostasis and continuous tissue perfusion via the cardiovascular circulation help to further reduce the temperature increase.

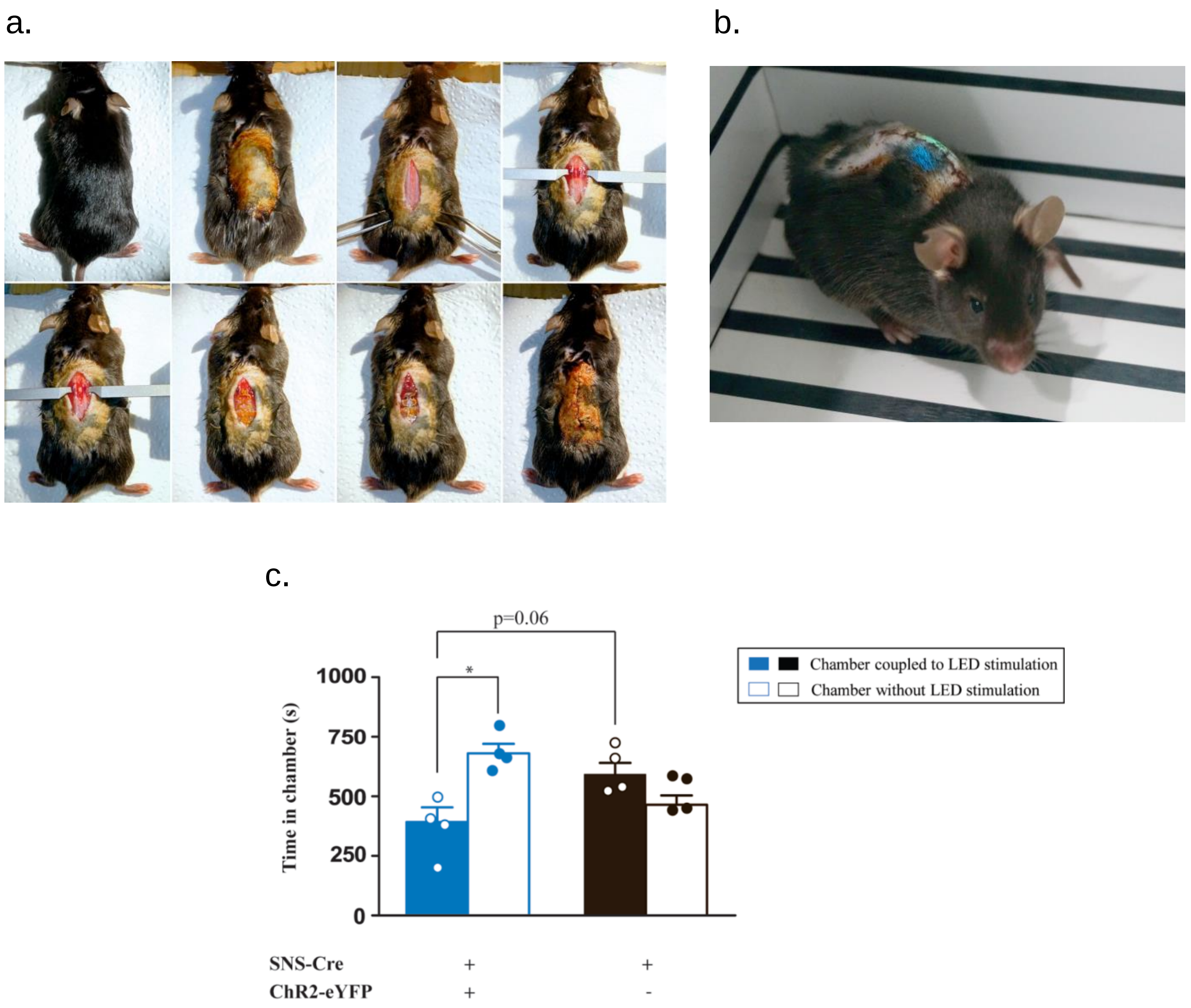

Fig.S9. *In vivo* spinal implantation of wireless device and place aversion behavior. a. Representative images of dorsal laminectomy and implantation procedure for wireless devices at the superficial dorsal horn of the spinal cord. An incision was made along the midline to expose the dorsal hump and spinal adaptors were used to secure the T13 vertebral disc. The disc was trimmed away to expose the L4-L5 lumbar spinal segment. The device was placed with the LED superficially over the spinal dura and sutured to the muscles. Tissue glue was applied and the skin was sutured. **b.** Awake freely moving mice implanted with a 2-LED device over the lumbar spinal cord. **c.** Place aversion experiment was performed with single blue-LED device implanted over the lumbar spinal cord of SNS-Cre transgenic mice expressing ChR2 transgene from the Rosa26 locus and without the ChR2 transgene, as indicated on x-axis. There was a significant interaction of genotype with time spent in either the stimulation coupled or non-coupled chamber (two-way ANOVA $F(1,12) = 18.7$, $p = 0.001$ (n = 4 mice for both groups). *, $p < 0.05$.

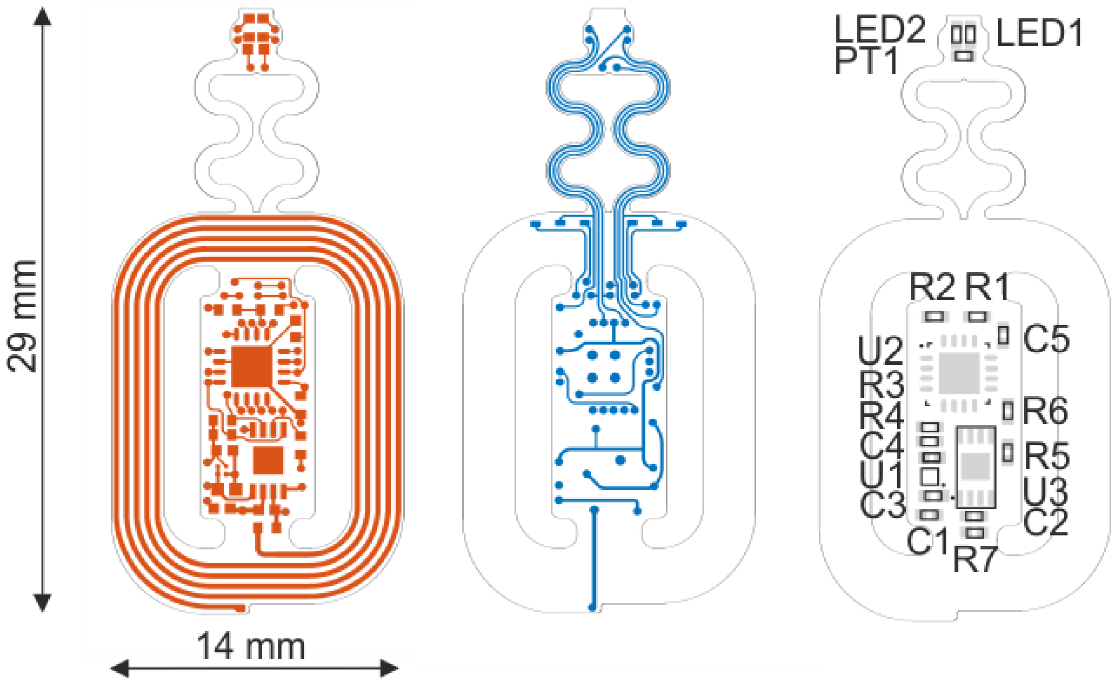

U1	LDO, TLV70528YFPT, 2.8 V, 200 mA,		
U2	M24LR64E-RMC6T/2		
U3	MKL03Z32VFG4		
C1	0402, 50 V	160 pF	±2 %
C2	0402, 50 V	14 pF	±2 %
C3	0402, 6.3 V	1 µF	±10 %
C4, C5	0402, 6.3 V	100 nF	±10 %
R1, R2	0402, 0.063 W	75 Ω	1 %
R3	0402, 0.063 W	100 kΩ	1 %
R4, R5	0402, 0.063 W	10 kΩ	1 %
R6	0402, 0.063 W	54.9 kΩ	1 %
R7	0402, 0.063 W	0 Ω	
PT1	NTC, ERT-J0EV104G, 0402	100 kΩ	2 %
LED1	LB QH9G-N100-35-1, 0402, blue 470 nm		
LED2	LT QH9G-Q200-25-2Z4Y, 0402, green 530 nm		

Fig.S10 Mask design of the 2-LED device and list of the components.

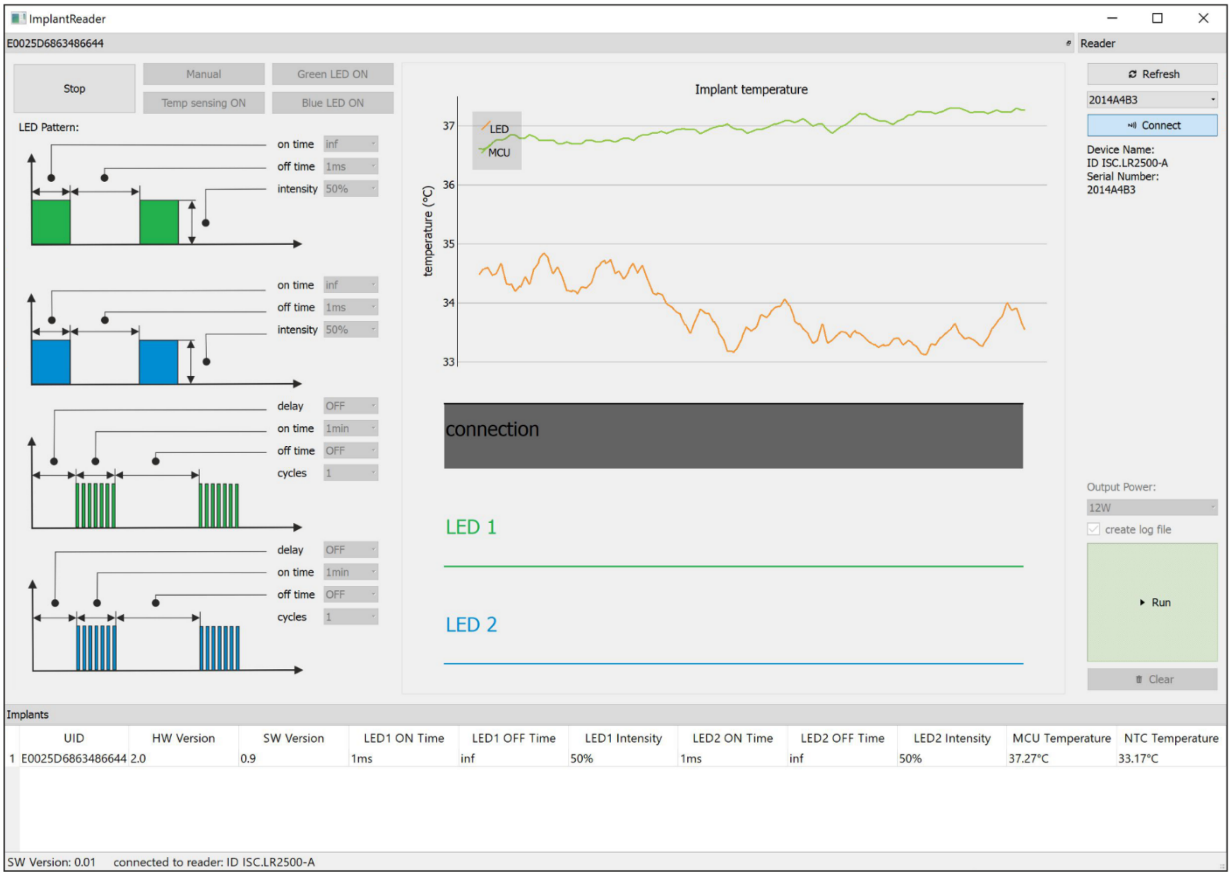

Fig.S11 PC application user interface designed to configure the wireless reader as well as to program and monitor the 2-LED devices.

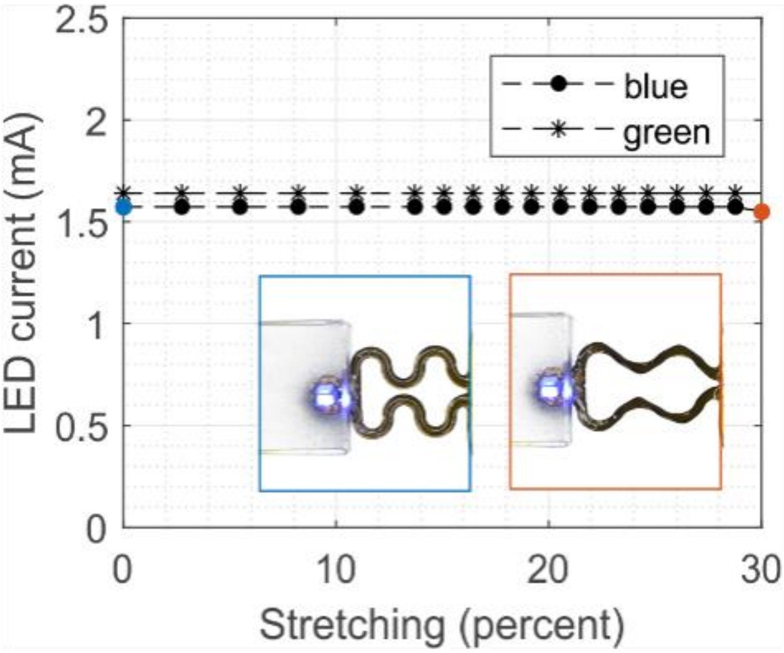

Fig.S12 Current of the blue and green LEDs when the interconnection is stretched up to 30 %.

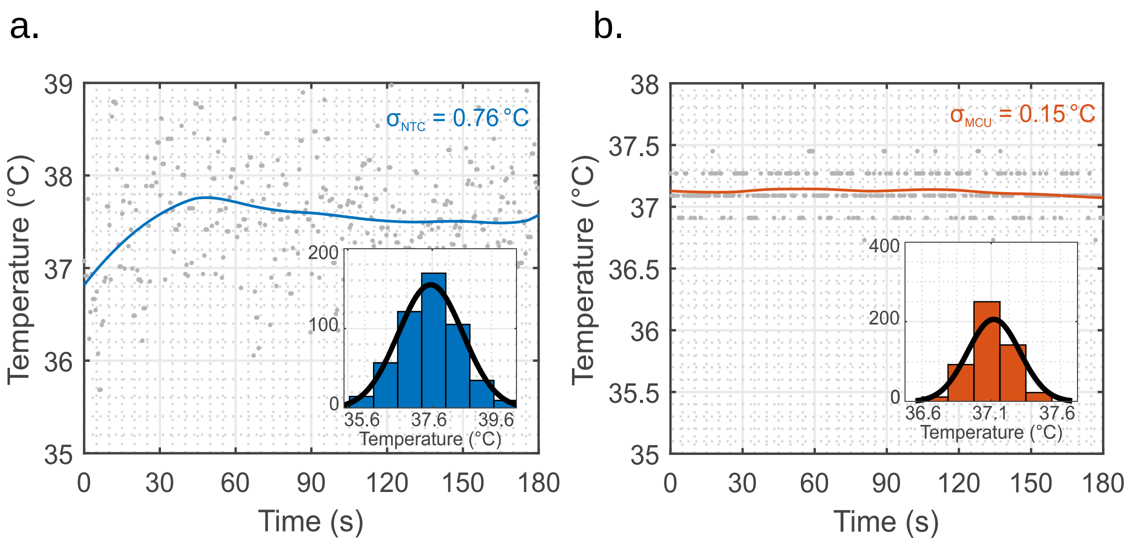

Fig.S13 In vivo measured and wireless transmitted temperature. a. Calibrated temperature measurement close to the LEDs after switching them on. The coupling of the external magnetic field in the analog domain of the device causes a standard deviation of 0.76 °C in idle state; **b.** Temperature measured at the microcontroller die.

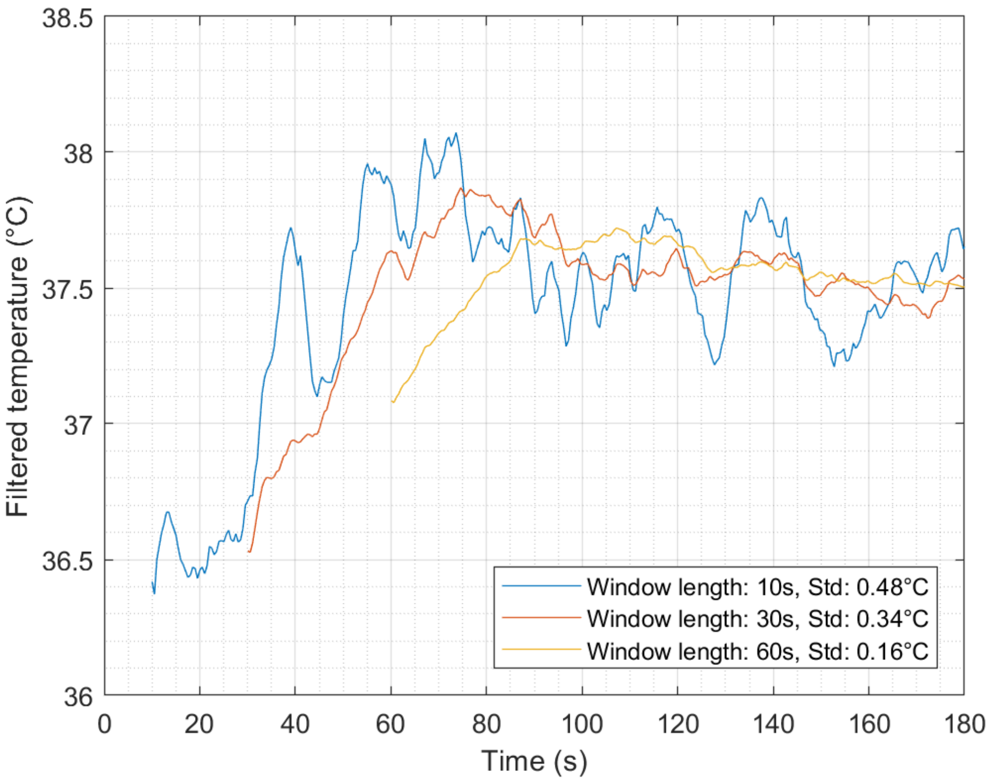

Fig.S14 Low pass filtering of wireless temperature measurement. The filtering is applied through an averaging over a moving window with various depth. The plots show the impact of the depth of the window on the standard deviation and on the response time. By increasing the depth the standard deviation decreases and the response time increases.

Table S1. Pairwise *post hoc* comparisons of latency measurements between different stimulations with R computational algorithm (Corresponding to Fig. 3d.)

Pairwise Comparisons	estimate	SE	df	t-ratio	p-value
Ipsilateral paw (470 nm) and Ipsilateral paw (470+530 nm)	-32.3	7.7	139	-4.2	0.0001
Ipsilateral paw (470 nm) and Contralateral paw (470 nm)	-46.8	7.7	139	-6.1	<0.0001
Ipsilateral paw (470+530 nm) and Contralateral paw (470 nm)	-14.5	7.7	139	-1.9	0.1473

[#]p value adjustment: Tukey method for comparing a family of 3 estimates
